# Thermally-Induced Shape-Memory Behavior of Degradable Gelatin-Based Networks

**DOI:** 10.3390/ijms22115892

**Published:** 2021-05-31

**Authors:** Axel T. Neffe, Candy Löwenberg, Konstanze K. Julich-Gruner, Marc Behl, Andreas Lendlein

**Affiliations:** 1 Institute of Active Polymers and Berlin-Brandenburg Center of Regenerative Therapies, Helm-holtz-Zentrum Hereon, 14513 Teltow, Germany; axel.neffe@hereon.de (A.T.N.); candy.loewenberg@web.de (C.L.); julich-gruner@helmholtz-muenchen.de (K.K.J.-G.); marc.behl@hereon.de (M.B.); 2 Institute of Chemistry, University of Potsdam, 14476 Potsdam, Germany

**Keywords:** shape-memory hydrogel, active polymer, biopolymer, mechanical properties, degradation

## Abstract

Shape-memory hydrogels (SMH) are multifunctional, actively-moving polymers of interest in biomedicine. In loosely crosslinked polymer networks, gelatin chains may form triple helices, which can act as temporary net points in SMH, depending on the presence of salts. Here, we show programming and initiation of the shape-memory effect of such networks based on a thermomechanical process compatible with the physiological environment. The SMH were synthesized by reaction of glycidylmethacrylated gelatin with oligo(ethylene glycol) (OEG) α,ω-dithiols of varying crosslinker length and amount. Triple helicalization of gelatin chains is shown directly by wide-angle X-ray scattering and indirectly via the mechanical behavior at different temperatures. The ability to form triple helices increased with the molar mass of the crosslinker. Hydrogels had storage moduli of 0.27–23 kPa and Young’s moduli of 215–360 kPa at 4 °C. The hydrogels were hydrolytically degradable, with full degradation to water-soluble products within one week at 37 °C and pH = 7.4. A thermally-induced shape-memory effect is demonstrated in bending as well as in compression tests, in which shape recovery with excellent shape-recovery rates R_r_ close to 100% were observed. In the future, the material presented here could be applied, e.g., as self-anchoring devices mechanically resembling the extracellular matrix.

## 1. Introduction

The large variety of functional groups present in biopolymers such as nucleic acids and proteins gives rise to thermodynamically preferred conformations of such biomacromolecules in an aqueous environment [1]. These conformations, typically referred to as secondary structures (short-range interactions), tertiary structures (overall conformation of a single chain), and quarternary structures (overall structure involving several polymer chains) are related to the non-covalent interactions between the functional groups and with water as well as the stereoelectronic effects of substituents along the chain. Protein conformations are generally much more studied and better understood than, e.g., polysaccharide conformations. The adoption of preferred conformations is sequence (primary structure)-dependent [2], and is therefore inherently included information. The conformation is associated with the biological function of the protein, such as specificity in biological interactions [3,4] or its mechanical performance [5,6]. As the conformations are based on non-covalent interactions, they can be altered, and, potentially, switched “on” or “off”, by changing the environment, such as pH, presence and concentration of ions or individual molecules, or temperature. This has inspired utilizing biopolymers as switching segments in shape-memory polymers (SMP). In addition, such biopolymer-based switching segments might impart degradability into the SMP [7,8], resulting in multifunctional materials [9]. Shape-memory hydrogels (SMH) are SMPs formed from hydrophilic polymers that are swollen in water [10,11]. These typically resemble the extracellular matrix in mechanical behavior as well as allow diffusion of nutrients so that SMH are of especial interest for biomedicine. 

While a metal coordination of carboxymethyl cellulose derivatives [12,13] contains bio-polymer-based switching segments, the so-formed temporary net points do not occur in nature in this way. In contrast, several proteins have been used as components or blueprints in shape-memory polymers, e.g., the switch between α-helical and β-strand conformations upon water uptake and at different strain was utilized in keratin-based materials [14]. The pH-dependent switching between an unordered and a β-strand conformation of a 20mer peptide was employed to form temporary net points in acrylate copolymers with grafted peptide groups [15]. Temperature-dependent adoption of β-strand-like structures has furthermore been shown for short, hydrogel-forming peptides containing aromatic groups, which consequently allowed shape fixation in SMP consisting of a double network [16].

Typical for collagen/gelatin systems is the association of three polymer strands into a triple helix, which typically dissociate upon heating to ~35–37 °C [17]. Biotechnologically produced ABA triblock peptides with collagen-like A blocks and lysine-containing B blocks that were covalently crosslinked with glutaraldehyde [18] showed a shape-memory effect after programming using the triple helix formation as temporary net points. This strategy, however, did not allow easy tailoring of the mechanical properties of the network. An interpenetrating double network consisting of a covalent polymeth-acrylate network and a physical gelatin network [19] likewise exploited the triple helix association, but required the synthetic polymer network for the shape-memory effect. Addition of graphene oxide incorporated near infrared sensitivity in that system [20]. Similar from the material design is a double network based on polymethacrylate and elastin [21], in which the temperature-dependent coiling of the protein chains is the basis for shape fixation. 

**Figure 1:**
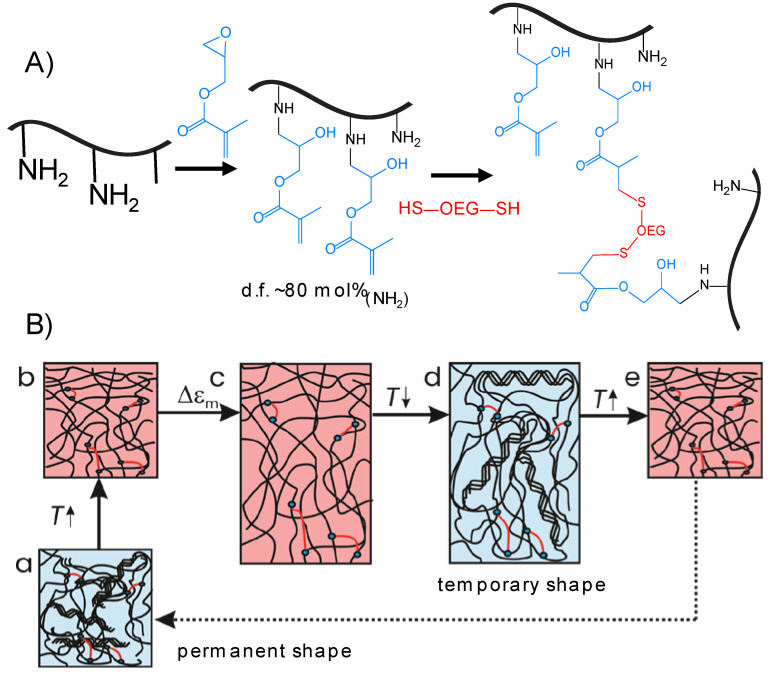
(**A**) Network formation is achieved in two steps: (i) functionalization of gelatin with glycidyl methacrylate; and (ii) crosslinking with an oligo(ethylene glycol) (OEG) α,ω-dithiol. (**B**) Molecular principle of the envisioned thermally-induced shape-memory effect of the investigated gelatin-based networks. The networks after synthesis at room temperature contain triple helices. (a) Heating leads to dissociation of the triple helices (b). Deformation at *T*_high_ (c) and subsequent cooling (d) fixes the temporary shape of the material by triple helices acting as temporary net points. (a→d) is the programming of the material. Subsequent heating leads to the dissociation of the temporary net points and readoption of the permanent shape, the shape-memory effect (e). Returning to room temperature re-establishes status (a) without further shape change.

We recently introduced a gelatin-based covalent network system that is formed by reacting glycidyl methacrylated gelatin (GMA-gelatin) with oligo(ethylene glycol) (OEG) α,ω-dithiols (Figure 1A) [22]. The approach to crosslink gelatin by a bifunctional crosslinker has in the past been demonstrated to be beneficial for effectively establishing a poly-mer network. As such a crosslinker can be varied in length and rigidity, network properties can be tailored without changing the crosslinking chemistry [23]. GMA-gelatin OEG networks are only loosely crosslinked so that the gelatin chains are flexible enough to adopt triple helices under favorable environmental conditions. In that study, we furthermore showed that the formation and dissociation of triple helices can be assisted by employing chaotropic or kosmotropic salts, and that in this way a salt-induced shape-memory effect could be realized. In comparison to the studies cited above, neither a biotechnological produced protein nor a second network is required; i.e., by simple polymer-analogous reactions the gelatin-based network could be established. The salt-based control of protein chain conformation in a hydrogel and exploiting it for a material function such as the shape-memory effect [22] is of fundamental interest, however, the required salt concentrations are likely not compatible with a biological environment. It is therefore of interest to investigate whether the shape recovery of gelatin/OEG-based SMH can be initiated by a stimulus compatible with the physiological environment, and whether changes in the composition of the networks influences properties and functions of the SMH.

Here, we explore in detail the chain organization (by wide angle X-ray scattering) and mechanical properties (by rheology and tensile tests at different temperatures) of hydrogels formed by reacting glycidyl methacrylated gelatin with OEG α,ω-dithiols of varied length and in different ratios. Furthermore, the hydrolytic degradation of such networks is described, and the shape-memory behavior using temperature as stimulus is explored and quantified in bending and compression tests. The network formation and the molecular principle of the shape-memory effect are depicted in Figure 1.

## 2. Results and Discussion

The synthesis of the gelatin-based network was performed by first functionalizing gelatin with double bonds by reaction with glycidyl methacrylate, and subsequent crosslinking the 20 wt.% GMA-gelatin solutions in water via thiol-Michael addition of OEG α,ω-dithiols (Figure 1A). The constitution of the networks was varied by the length of the OEG crosslinkers (*M*_n_ = 1000, 1500 or 3400 g·mol^−1^) and ratio between thiol and methacrylate groups (0.75, 1, 2, or 3). The networks are in the following referred to as G20_OEG*y*(*z*), with *y* = number average molar mass *M*_n_ of the OEG crosslinkers and *z* the thiol:methacrylate molar ratio. The gel content *G,* which is a measure of network formation, and the volumetric swelling *Q* of the G20_OEG1000(*z*) and G20_OEG3400(*z*) networks has been reported before [22] and is given in detail for the G20_OEG1500(*z*) in Supporting Information Table S1. As a general trend, a reduction of *G* with increasing length of the crosslinker as well as with increasing *z* was observed, though there were only small differences between G20_OEGy(0.75) and G20_OEGy(1) samples. The decrease of *G* with the length of the crosslinker may reflect the somewhat lower reactivity of thiol end groups on larger, potentially coiled OEG, which may have led to a steric hindrance of the reactive group. Increasing *z* leads to an increase of grafted OEG groups in comparison to OEG crosslinks. This may result in washing out not only of unreacted OEG, but also gelatin-*g*-OEG when determining *G*. Furthermore, when larger amounts of thiols are present, in addition to the Michael-type addition to the methacrylate groups thioester formation also may take place that would result in the formation of soluble OEG dimethacrylate thioesters (see Supporting Information Figure S1). *Q* increased with *z* as well as with OEG length, showing that the network density decreases upon these changes in composition. Temperature did not have an influence on *Q*. This is relevant, as otherwise the later-discussed shape recovery could partially be explained by a swelling effect upon heating, which is not the case here.

**Figure 2:**
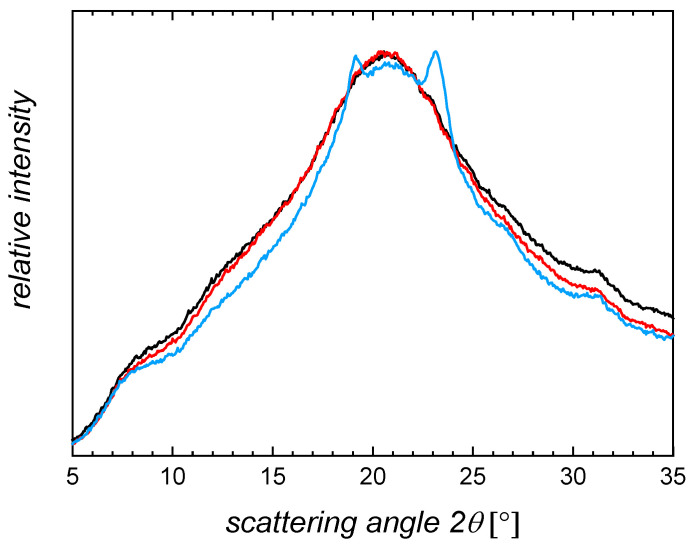
Wide angle X-ray scattering (WAXS) curves of dried G20_OEG1000(1) (black), G20_OEG1500(1) (red), and G20_OEG3400(1) (blue) networks at ambient temperature.

Dried gelatin network samples were investigated by wide angle X-ray scattering (WAXS, Figure 2 and Supporting Information Figure S2) to characterize the ability of the gelatin chains to adopt triple helices. Gelatin samples typically show three peaks in a WAXS analysis. Two narrow peaks appear at 2θ = 7.5° and 2θ = 33°, which correspond according to Bragg’s law to distances of 1.1 nm (intermolecular distance between collagen triple helices, and indicative of triple helicity) and 0.29 nm (distance of amino acidic residues along the left-handed single helices) [24]. A third, broad peak signifies the amorphous chain organization (2θ~21°, d~0.45 nm). In all investigated samples, a peak at 2θ = 7.5° was observed and hence triple helices, which are the envisioned prerequisite for shape fixation of temporary shapes, were formed. By relating the area under the peak corresponding to the triple or single helix to the overall area under the curve, an index for the single and triple helical content can be calculated (Supporting Information Table S2). It should be noted that these indices are not an absolute value of helical content, as even compounds nearly completely adopting a triple helical conformation also show scattering resulting in an amorphous halo. However, the index can be employed to discuss changes in the ability or tendency to form triple helices [23,25] and the association of them to fibrils. Here, the triple- and single helical indices (*X*_TH_ and *X*_SH_) were higher for the G20_OEG3400(*z*) networks (5.6–6.1 ± 0.5%) compared to the networks formed with shorter OEG (3.9–4.6 ± 0.2%). This may translate into a higher shape-fixity ratio for the G20_OEG3400(*z*) when programming samples for an SME compared to the other networks. No changes were observed for different thiol:methacrylate molar ratios. The increased tendency for adopting triple helices with increasing chain length of the crosslinker may be explained by the larger degree of freedom for individual chains in such networks.

**Figure 3:**
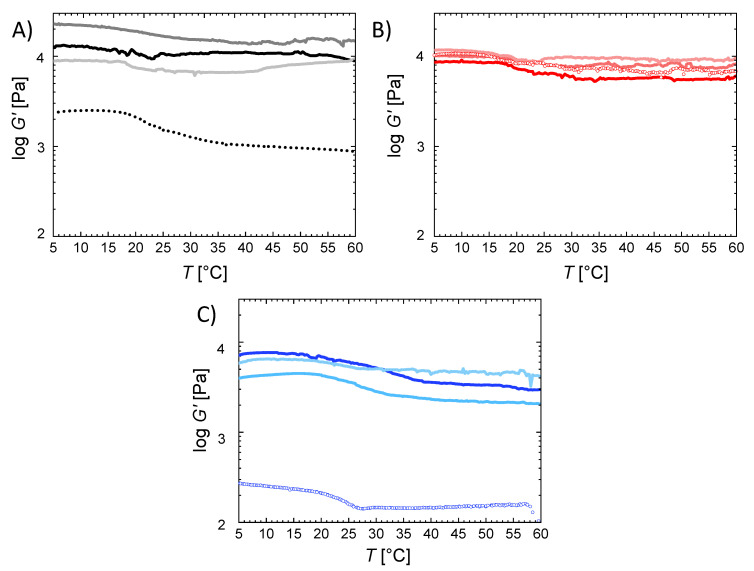
Storage modulus G‘ of (**A**) G20_OEG1000(z), (**B**) G20_OEG1500(z) and (**C**) G20_OEG3400(z) hydrogels at different temperatures. z = 0.75 (**^__ __ __^**), 1 (**^__ __ __^**), 2 (**^__ __ __^**), and 3 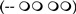.

**Figure 4:**
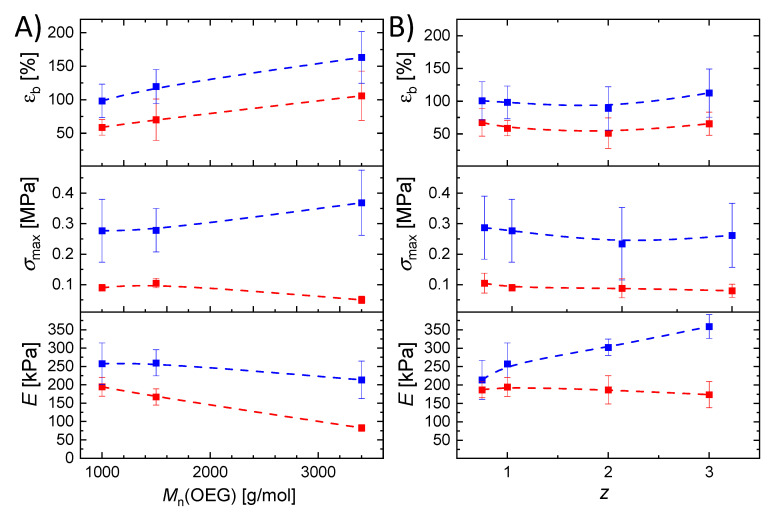
Elongation at break (*ε*_b_), tensile strength (*σ*_max_), and Young’s modulus (*E*) of the gelatin-based hydrogels under variation of OEG number average molar mass (**A**), or thiol:alkene molar ratio of the G20_OEG1000(z) hydrogels (**B**), determined in tensile tests at 4 °C (blue) and 55 °C (red) in a water chamber. The lines are added as a guide to the eye.

The mechanical properties of the hydrogels were evaluated in two setups: by rheology (Figure 3) and by tensile tests (Figure 4). The equilibrium swollen hydrogels exhibited storage moduli *G*′ between 270 ± 100 Pa and 22.8 ± 1.2 kPa. A decrease of *G*′ was observed with increasing chain length of the crosslinker and increasing *z*, mirroring the effects and rationale also observed and given for *Q*. Furthermore, *G*′ decreased when increasing the temperature, with a median transition temperature *T*_trans_ = 25 ± 3 °C determined in oscillation. In an additional experimental setup, the transition was investigated in rheological compression experiments (Supporting Information Figure S3) and was in this setup 28 ± 2 °C. This observation demonstrated the temperature sensitivity of the mechanical properties and can be attributed to the disaggregation of gelatin triple helices above *T*_trans_, which consequently reduced the net point density. At temperatures below *T*_trans_ the gelatin chains self-assemble to helices acting as additional net points. Consequently, at temperatures below *T*_trans_ (*T*_low_) the hydrogels contain permanent net points, formed by covalent crosslinking, and additional temporary net points, formed by helicalization of the gelatin, while at temperatures above *T*_trans_ (*T*_high_) only the permanent net points contributed to the network strength. The transition temperature is notably lower than in non-crosslinked gelatin (*T*_trans_ ~ 35 °C) [26], which suggests that the length of the formed triple helices in the networks studied here is quite limited.

With increasing OEG chain length the difference between the initial plateau of *G*′ at *T*_low_ and the plateau of *G*′ at *T*_high_ was increasing. This corresponds to the finding from the WAXS studies that these hydrogels were more prone to adopt triple helical conformations, and therefore are understood to have a higher percentage of temporary net points that are cleaved at *T*_high_ compared to the studied networks with low OEG length. This observation was in agreement with the literature, revealing that the number of physical net points formed by self-organization into helices influences the difference between *G*′(*T*_low_) and *G*′(*T*_high_) [27].

The uniaxial tensile tests (values in Figure 4, exemplary curves in Supporting Information Figure S4) were performed on swollen samples under water at two different temperatures (*T*_low_ = 4 °C and *T*_high_ = 55 °C), with covalent as well as temporary net points due to triple helicalization expected at *T*_low_, and an amorphous network with covalent net points at *T*_high_. The hydrogels accordingly showed increased Young’s moduli *E,* elongation at break *ε*_b_, and tensile strength *σ*_max_ at *T*_low_ compared to *T*_high_. At *T*_high_, the helices disaggregated, resulting in less resistance against uniaxial stretching compared to *T*_low_*. ε*_b_ was between 51 ± 23% and 163 ± 35%. Lowest *ε*_b_ values were observed for hydrogels with *z* = 1 and *M*_n,OEG_ of 1000 g⋅mol^−1^, indicating the highest degree of crosslinking. Additionally, *ε*_b_ was increasing with increasing OEG chain length. The hydrogel’s *σ*_max_ was between 0.05 ± 0.01 MPa and 0.36± 0.10 MPa, and was lower at *T*_high_ (0.05–0.1 MPa) than at *T*_low_ (0.26–0.36 MPa). A comparison of *σ*_max_ values at constant temperature showed almost no correlation of OEG chain length or amount and *σ*_max_, as the obtained data were all within the margin of error. However, a minor trend could be shown at *T*_low_, at which an increase of *σ*_max_ was correlating with an increasing OEG length. The Young’s modulus *E* was in the range of 83 ± 25 kPa to 356 ± 32 kPa. An increase in OEG length led to a decrease in *E*, which was due to an increase in mesh size and therefore increased flexibility of the polymer chains within the network. Indeed, the increase in OEG content showed different effects on *E*, depending on the temperature. At *T*_low_, *E* increased with increasing *z*, attributed to the increase of dangling ends and consequently a decrease in number of permanent net points. This furthermore increased the ability of helix formation acting as temporary net points resulting in an increase of the overall net point density, which led to an increase in material resistance against uniaxial deformation. At *T*_high,_ no influence of OEG content on *E* was observed, as only the permanent net points contributed to the mechanical properties.

**Figure 5:**
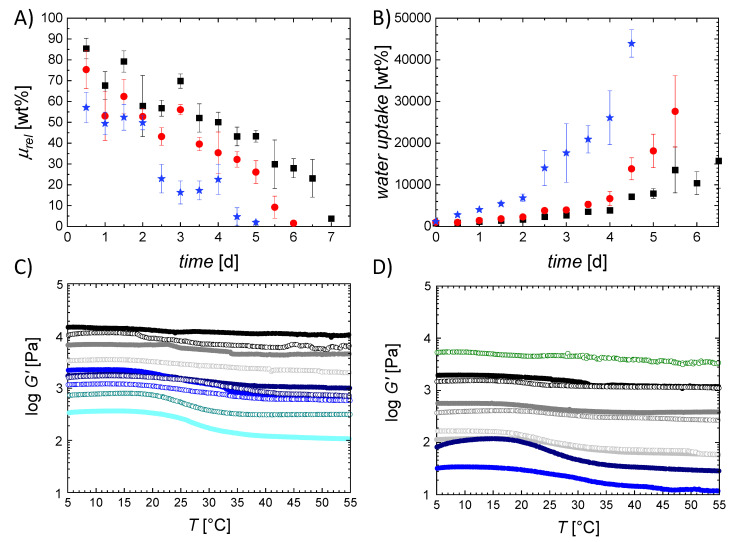
Hydrolytic degradation of G20_OEGy(z) hydrogels at 37 °C and pH = 7.4: (**A**) remaining mass *µ_rel_* and (**B**) water uptake *H* over time. G20_OEG1000(1): black, G20_OEG1500(1): red, G20_OEG3400(1): blue. C/D) Rheological behavior of G20_OEG1000(1) (**C**) and G20_OEG3400(1) (**D**) hydrogels after 0.5 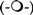, 1(-●-), 1.5 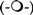, 2(-●-), 2.5 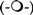, 3 (-●-), 3.5 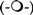, 4 (-●-), 4.5 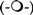, 5 (-●-), 5.5 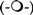, 6.5 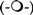, and 7 (-●-) days of hydrolytic degradation.

While the OEG chains themselves are not hydrolytically degradable, they are connected to the gelatin backbone by ester bonds, which can be hydrolytically cleaved. This is also true for the amide bonds of the gelatin chains, which are however more stable to hydrolysis than esters. The degradation of the network was studied by quantifying the mass loss, the change of water uptake, as well as the change of mechanical properties of the G20_OEGy(1) networks during degradation (Figure 5 and Supporting Information Figure S5).

The rate of hydrolytic degradation was dependent on the chain length of the crosslinker. An increase of the chain length led to an increase in mass loss and water uptake during degradation, attributed to the increasing mesh size and hydrophilicity of the hydrogel, both contributing to a faster diffusion of water into the network and, potentially, also to a faster release of fragments. The change of mechanical strength of the hydrogels was investigated by rheology recording the storage modulus in temperature-dependent oscillation tests. *G*’ strongly depended on the degradation time and was decreasing by about two orders of magnitude during degradation. The effect was more evident for increased chain length of the OEG crosslinker, as rationalized above regarding the mass loss and water uptake. Furthermore, the difference between the plateau at *T*_low_ and *T*_high_ increased with increasing degradation time. This is likely due to the decreasing net point density and resulting increase in mesh size during degradation, which increases the flexibility of the polymer chains. At increased degradation time the amount of helices was increased, which is why a sharper transition can be observed, compared to the non-degraded hydrogels. 

In comparison to hydrolytic degradation experiments of other gelatin-based hydrogels, where the complete degradation took more than six weeks [28,29], the GMA–OEG hydrogels showed a faster degradation. This suggests that it is not the hydrolysis of the main chains, but rather the hydrolysis of the crosslinker that is here the rate determining step of the degradation. While the oligoethers are not hydrolytically degraded, the ester bonds from the original glycidyl methacrylate group are likely the primary point of attack. A further experiment supports this hypothesis: an SDS-polyacrylamide gel electrophoresis (PAGE) was conducted from the gelatin starting material and the partially degraded gelatin-based networks. In the PAGE, no gelatin fragments were observed (Supporting Information Figure S6). It can be furthermore speculated that in systems with a high thiol:methacrylate molar ratio, and hence free thiol groups, degradation may be assisted by ester–thioester exchange reactions according to Supporting Information Figure S1.

**Figure 6:**
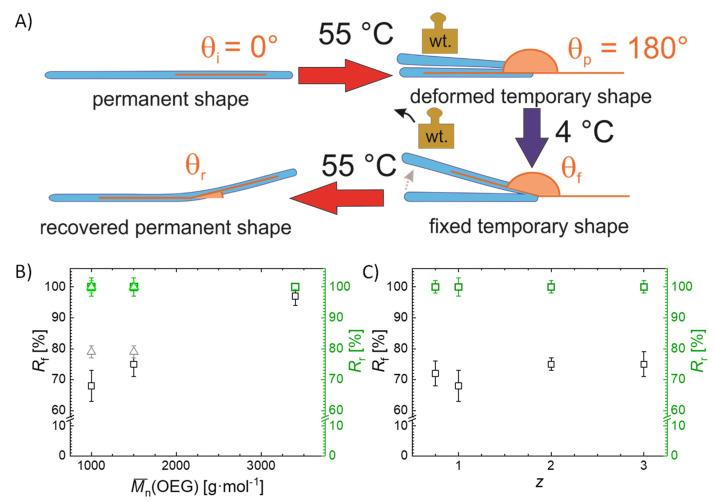
Shape-memory properties determined by bending experiments with temperature as stimulus. (**A**) Schematic illustration of the experimental setup for the determination of shape-fixity and shape-recovery ratios. (**B**) *R*_f_ and *R*_r_ as a function of OEG chain length for G20_OEGy(z) hydrogels. (**C**) *R*_f_ and *R*_r_ as a function of OEG content for G20_OEG1000(z) hydrogels. (□: z = 1, Δ: z = 2).

The principle of characterization of the shape-memory properties by bending experiments is depicted in Figure 6A. The hydrogels were cut into stripes and bent by 180° after heating above *T*_trans_ (to 55 °C). The deformed hydrogel stripes were cooled below *T*_trans_ (to 4 °C) for 8 h, while keeping the applied deformation to allow helicalization of the gelatin chains acting as physical net points to fix the temporary shape. Then, the applied deformation was removed obtaining the temporary shape. The permanent shape was recovered upon heating above *T*_trans_. The hydrogels exhibited *R*_f_ values between 68 ± 5% and 97 ± 3%, where *R*_f_ was increasing with increasing OEG chain length (Figure 6B). This observation was related to the higher degree of triple helix formation fixing the temporary shape as temporary net points. Additionally, hydrogels with *z* = 1 exhibited the lowest *R*_f_ (Figure 6C). Because the *z* = 1 networks have the highest net points density of the investigated hydrogels, the formation of helices acting as net points for the fixation of the temporary shape is here the lowest. Conclusively, the ability for the fixation of the temporary shape directly correlated to the amount of helices in the network. All hydrogels exhibited excellent *R*_r_ of 100%, independent of the OEG chain length and OEG content, revealing that the recovery of the permanent shape is independent of the helix content.

**Figure 7:**
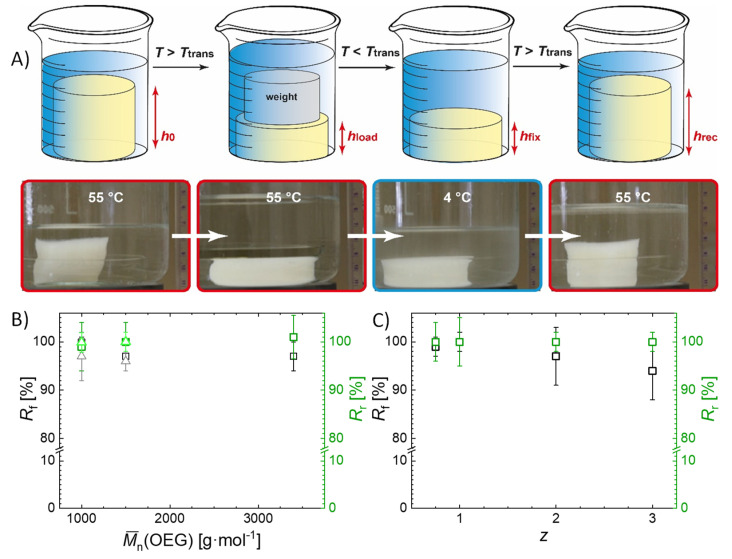
Shape-memory properties determined by compression experiments with temperature as stimulus. (**A**) Schematic illustration and images of the experimental setup. (**B**) *R*_f_ and *R*_r_ as a function of OEG chain length for G20_OEGy(z) hydrogels. (**C**) *R*_f_ and *R*_r_ as a function of OEG content for G20_OEG1000 (z) hydrogels. (□: z = 1, Δ: z = 2).

A second method for the determination of the shape-memory properties was compression testing (Figure 7A). Here, the hydrogels were compressed to 35–50% of their initial height at *T* = 55 °C, i.e., above *T*_trans_ of the gelatin chains. Afterwards, the hydrogels were cooled to *T* = 4 °C for at least 8 h. By increasing the temperature again above *T*_trans_, the initial shape was recovered. Since volumetric shrinking or swelling could hinder or distort the observation and quantification of the SME, the volume of the hydrogel samples in the permanent shape (*V*_perm_ = 46 ± 3 cm^3^) and in the temporary shape (*V_temp_* = 49 ± 3 cm^3^) were determined by measuring the outer dimensions of the gels. As the values were within the margin of error, the volume of the samples is understood to have no major influence on the SME, and shape change only occurs due to entropy-elastic recoiling, i.e., an SME. This corresponds to the observation that the degree of swelling *Q* does not change with the temperature for the investigated samples, furthermore supporting the understanding that a true shape-memory effect was observed. In compression mode, the hydrogels exhibited excellent *R*_f_ (from 94 ± 6 to 100 ± 5%) and *R*_r_ values (100 ± 5%), and no influence of the hydrogel composition on the shape-memory properties was observed (Figure 7B,C). In contrast to the *R*_f_ values determined by bending tests, the *R*_f_ values determined by compression tests were higher. This observation can be related to the surface-to-volume ratio of the sample. In compression tests, the whole sample could contribute to the shape fixation, while in bending experiments only the small part of the bent area was involved in the shape fixation. While a complete shape recovery was observed for all samples in both SME setups, it should be noted that the determined *R*_f_ may potentially reduce over time because of creep, which however has not been investigated in this study.

In the design of thermally-induced shape-memory hydrogels, one can distinguish between the thermal transition temperature associated with the switching domain (*T*_trans_) and the switching temperature (*T*_sw_), at which the shape change is observed. These can be different because of the influence of heat transfer and dissipation, the water diffusion rate, as well as geometric effects of the investigated sample. In the current example, *T*_trans_ as determined by rheology (25 ± 3 °C in oscillation, 28 ± 2 °C in compression) was excellently correlating with *T*_sw_ (26 ± 3 °C). This means that the shape recovery is compatible with a physiological environment. While the shape recovery was quantified at *T*_high_ = 55 °C, a full recovery is also expected at 37 °C, though with a lower speed of recovery. Supporting Information Figure S7 and the Supporting Information Video V1 show the programming of a hydrogel strip as a helix and the recovery of the permanent form.

## 3. Materials and Methods

### 3.1. Materials

Gelatin (type A, 200 bloom), and coomassie blue were purchased from Fluka (Munich, Germany). Glycidyl methacrylate, OEG1000, OEG1500, OEG3400, TNBS, sodium chloride, sodium thiocyanate, sodium bicarbonate, ethanol, hydrochloric acid, diethyl ether, acetic acid, dichloromethane, methanol, THF, DMSO-D6, D_2_O, and SDS-PAGE sample buffer were obtained from Sigma Aldrich (Munich, Germany). Sodium carbonate, tris(hydroxymethyl)-aminomethane, glycine, SDS, ammonium sulfate, sodium sulfate, magnesium chloride, disodium-hydrogenphosphate, and sodium dihydrogenphosphate were purchased from Merck (Darmstadt, Germany). All solvents and reagents were used without further purification.

### 3.2. GMA-Gelatin

Gelatin (10 wt.%) was dissolved in bicarbonate buffer (0.05 M, pH 9.6: 1.59 g sodium carbonate and 2.93 g sodium bicarbonate in 1 L water) at 50 °C. GMA was added with a dropping funnel, and the reaction was stirred at 50 °C for 3 h. The product was precipitated in 5 vol. eq. ethanol at room temperature (RT). The GMA-gelatin was cut into smaller pieces that were dried at 40 °C under reduced pressure. The degree of functionalization was determined by 2,4,6-Trinitrobenzenesulfonic acid (TNBS) assay.

### 3.3. TNBS Assay 

Gelatin or GMA-gelatin (11 mg) was dissolved in 1 mL of 4 wt.% NaHCO_3_ (pH 8.5) containing 1 mL of 0.5 wt.% TNBS. The reaction mixture was shaken mildly at 40 °C for 4 h; 3 mL 6 M HCl was added and the mixture was heated at 120 °C for 1 h. After cooling to 25 °C, 5 mL of water was added. The mixture was extracted three times with 20 mL ethyl ether. A 5 mL aliquot of the aqueous phase was heated for 20 min in a hot water bath to evaporate the residual ether. The aliquot was diluted to 20 mL with water and the absorbance was measured at 346 nm. All experiments were performed in triplicates and read against a blank that was prepared by the same procedure while adding the HCl before the addition of TNBS. The degree of functionalization (DF) was calculated according Equations (1) and (2), where A is the measured absorbance at 346 nm, V the volume of the aliquot (0.02 L), 1.46 × 10^4^ the absorption coefficient of the 2,4,6-trinitrophenyl derivative, *l* the cell path length (1 cm), m the sample weight (11 mg), and n_NH2°_ the amount of free amino groups before functionalization (3.48 × 10^−4^ mol/g).

(1) $$n_{NH_{2}}=\frac{2\cdot A\cdot V}{1.46\times 10^{4}\cdot l\cdot m}$$

(2) $$DF=100-\frac{n_{NH_{2}}}{n_{NH_{2}^{0}}}\times 100\%$$

### 3.4. Network Synthesis

The networks were synthesized by dissolving 1 g GMA-gelatin in 5 mL water and addition of OEG dithiols with varying molar masses (*M*_n_ = 1000, 1500, or 3400 g∙mol^−1^) dissolved in 5 mL of water (20 wt.%). After stirring the reaction mixture for one minute, the mixture was cast into a Petri dish, followed by drying at 40 °C overnight. 

### 3.5. Gel Content

The gel content *G* was determined by comparing the dry weight of the sample after extraction (24 h at r.t., changing the water eight times) (m_extr_) with the dry weight before extraction (m_iso_) according to Equation (3). These extracted samples were used in the further experiments.

(3) $$G=100\%\cdot \frac{m_{extr}}{m_{iso}}$$

### 3.6. Rheology

Rheological investigations were performed on equilibrium swollen samples on a Haake Rheowin Mars II or Haake Rheowin Mars III (Thermo Scientific, Karlsruhe, Germany) using a 20 mm plate–plate geometry. A solvent trap was placed above the sample to avoid water evaporation. Temperature ramp measurements were performed on three replicas with a controlled stress of 4 Pa and at a frequency of 1 Hz, as the systems were in the linear viscoelastic range under these conditions. The heating rate was 1 K·min^−1^. A constant force of 1 N was used on the as-prepared hydrogels, while a constant force of 0.1 N was applied in the hydrolytically partially degraded networks. In rheological compression experiments for the determination of *T*_trans_, samples were compressed repeatedly to 50% of height and relaxed again, increasing the temperature from 10 to 50 °C in increments of 2 °C and registering the required force for the compression.

### 3.7. Tensile Tests

The mechanical properties were investigated with a tensile tester Zwick ZP 99 (Zwick GmbH, Ulm, Germany), equipped with a force transducer of maximum 20 N in a water chamber (distilled water) at *T*_low_ (4 °C) and *T*_high_ (55 °C). The measurements were carried out with minimum 6 repetitions on dog bone-shaped samples (ISO 527-2/1BB, 30 × 2 mm) after determination of the sample thickness, registering engineering stress *σ* and strain *ε*. The samples were elongated with a speed of 5 mm·min^-1^ using a preforce of 5 mN. *E* was calculated manually as the slope of the initial linear segment of the tensile test curve, typically at strains <2%. Before the measurement, all samples were swollen to equilibrium in distilled water. To delete the thermal history, the samples were heated up to 55 °C for 10 min, and cooled for 8 h at 4 °C to allow helicalization.

### 3.8. Wide Angle X-ray Scattering (WAXS)

WAXS measurements were performed on dried samples on a Bruker D8 Discover with a 2D-detector from Bruker AXS (Karlsruhe, Germany) using 3 replicates for each sample. WAXS images were collected from gelatin films in transmission geometry with a collimator-opening of 0.8 mm at a sample-to-detector distance of 15 cm. The X-ray generator was conducted at a voltage of 40 kV and a current of 40 mA, with Cu-Kα radiation of a wavelength of λ = 0.154 nm, covering a 2θ range of ~3 to 37°. An exposure time of 300 s per frame was used, and an empty sample holder was subtracted as background. The determination of the index of single and triple helicity (Χ_TH_, Χ_SH_) was determined according to Equations (4) and (5):

(4) $$X_{TH}=\frac{100\cdot A_{TH}}{A_{amorph}}$$

(5) $$X_{SH}=\frac{100\cdot A_{SH}}{A_{amorph}}$$

where *A*_TH_ = area under the peak related to the triple helices, *A*_amorph_ = area of the amorphous peak, and *A*_SH_ = area under the single helix peak. The margin of error was obtained in a manual fitting, wherefore it was quite small. In the following, the mean value of 6 fittings ± s.d. is given. Instrumental contributions to the peak broadening were negligible. Primary data were corrected for geometric distortions (spatial correction) and the center and the distance of the sample to detector were calibrated utilizing the corundum standard.

### 3.9. Determination of Shape-Memory Properties

For the determination of the strain-fixity ratio (*R*_f_) and the strain-recovery ratio (*R*_r_) by bending, the equilibrium swollen hydrogels were cut into stripes (5 × 1 cm). For programming, the samples with an initial angle *θ*_i_ (= 0° for the first cycle) were heated to 55 °C and bent with an angle *θ*_p_ = 180° in the middle of the stripe and subsequently cooled at 4 °C for 12 h while keeping the deformation. The temporary shape was obtained after release of the applied load and the angle of the fixed shape (*θ*_f_) was determined. The programmed samples were heated to 55 °C to recover the permanent shape. Finally, the angle of the recovered shape was measured (*θ*_r_). For the determination of the angles between the segments of the folded stripes, photographs of the stripes were recorded and analyzed by ImageJ. *R*_f_ and *R*_r_ were calculated according to Equations (6) and (7) as the average from five cycles:

(6) $$R_{f}=\frac{\theta_{f}-\theta_{i}}{\theta_{p}-\theta_{i}}\;\cdot 100\%$$

(7) $$R_{r}=\frac{\theta_{f}-\theta_{r}}{\theta_{f}-\theta_{i}}\;\cdot 100\%$$

For the determination of the shape-memory properties in compression tests, the equilibrium swollen hydrogel cylinder (diameter of 4 cm and height of 3 cm (h_0_)) were heated to 55 °C and compressed to about 35–50 height % (*h*_load_). The hydrogels were immediately cooled to 4 °C for at least 8 h while keeping the compression, to enable the helicalization of the gelatin chains. Afterwards, the applied load was released and the height of the fixed shape was measured (*h*_fix_). The temperature was raised to 55 °C, the initial shape was recovered, and the height of the recovered shape was measured (*h*_rec_). *R*_f_ and *R*_r_ were calculated from 5 cycles according Equations (8) and (9).

(8) $$R_{f}=\frac{h_{fix}-h_{0}}{h_{load}-h_{0}}\;\cdot 100\%$$

(9) $$R_{r}=\frac{h_{rec}-h_{fix}}{h_{0}-h_{fix}}\;\cdot 100\%$$

### 3.10. Degradation

For degradation studies, the swollen hydrogels were punched in circular shape with a diameter in the wet state of 0.5 cm or 2 cm (depending on the analysis) and dried until constant weight was achieved. The 2 cm discs were used for the determination of the thermomechanical properties using rheology, while the 0.5 cm discs were used for determination of water uptake (*H*) and mass loss. Mass loss studies were performed with 6 repetitions per time point, while rheology was performed twice per time point. The mass of the dry discs before degradation was determined (*m*_0_). Afterwards, the samples were incubated in 15 mL PBS buffer at 37 °C in a shaker, and samples were removed and analyzed at the predetermined time points (typically every 12 h up to a maximum time of seven days). Subsequently, the samples were washed with 50 mL water for 8 h, changing the water each hour to remove the salts of the buffer solution and the weight of the swollen degraded sample was determined (*m*_sw-deg_). The partially degraded samples were dried until constant weight was achieved and the dry weight after degradation was determined (*m*_deg_). *H* was calculated in accordance with Equation (10), and the remaining mass (*μ_rel_*) according to Equation (11), and the change in thermomechanical properties was evaluated as a function of degradation time.

(10) $$H=\frac{m_{sw-deg}-m_{deg}}{m_{deg}}\;\cdot 100\%$$

(11) $$\mu_{rel}=100-\frac{m_{0}-m_{deg}}{m_{0}}\;\cdot 100\%$$

## 4. Conclusions

In networks based on the reaction of GMA-gelatin with OEG α,ω-dithiols, the conformational freedom of gelatin chains is large enough so that triple helices are formed below the system-specific transition temperature *T*_trans_ = 25±3 °C. The ability to form such triple helical regions increases notably with the *M*_n_ of the crosslinker, and there was also a tendency for higher triple helicalization with increasing dissimilarity between the amount of thiol and methacrylate groups. Triple helicity could be shown directly by WAXS as well as indirectly in temperature-dependent mechanical tests below and above *T*_trans_. The storage moduli of the studied hydrogels could be varied by 1–2 orders of magnitude according to the composition, while Young’s moduli and elongation at break varied by up to 50% between different compositions. The hydrogels were hydrolytically degradable, and mass loss as well as change of water uptake as well as rheological behavior was notable within a few days, which is remarkably faster compared to other gelatin-based hydrogels. This can be rationalized by the fact that the here investigated systems were only loosely crosslinked, and that bond cleavages took place primarily in the crosslinker rather than in the gelatin main chains. The ability of the systems to form triple helices could be used to enable a thermally-induced shape-memory effect compatible with physiological temperatures, which was shown in bending as well as in compression tests. Fixation of the temporary shape was based on the formation of triple helices as temporary physical net points. After disaggregation of the triple helices upon heating, entropy-elastic recoiling and shape recovery with excellent shape recovery rates *R*_r_ was observed. Additionally, the shape of the sample affected the ability for form fixation, with compressed samples having much higher *R*_f_ values than the bended samples. In the future, the here discussed shape-memory hydrogels may be of interest as multifunctional, actively-moving polymers that could be used in biomedicine, e.g., for self-anchoring devices.

## Data Availability

The data that support the findings of this study are available from the corresponding author upon reasonable request.

## Supplementary Materials

The following are available online at https://www.mdpi.com/article/10.3390/ijms22115892/s1, Figure S1: Thioester formation by reaction of a thiol with a glycidylmethacrylate group on GMA-gelatin as side reaction increasing in importance with increasing amount of thiol, Figure S2: WAXS spectra of (A) G20_OEG1000(z), (B) G20_OEG1500(z), and (C) G20_OEG3400(z) networks. z = 0.75 (__ __ __), 1 (__ __ __), 2 (__ __ __), and 3 (-- -- --), Figure S3: Determination of the transition temperature by rheological compression tests. A) G20_OEG1000(1), B) G20_OEG1000(2), C) G20_OEG3400(1), D) G20_OEG3400(2), Figure S4: Tensile tests of G20_OEG1000(1) samples at 4 °C (A) and 55 °C (B). σ = stress, ε = strain, Figure S5: Rheological behavior of G20_OEG1500(1) hydrogels after 1 (-λ-), 1.5 (-μ-), 2 (-λ-), 2.5 (-μ-),3.5 (-μ-), 4 (-λ-), 4.5 (-μ-), 5 (-λ-), 5.5 (-μ-), 6.5 (-λ-), and 6.5 (-μ-) days of hydrolytic degradation at 37 °C at pH = 7.4, Figure S6: SDS-PAGE of the molar masses of the degradation products at t = 8 d: (1) standard, (2) G20_OEG1000(1), (3) G20_OEG1500(1), (4) G20_OEG3400(1). No larger gelatin fragments are observed, which suggests that hydrolysis primarily starts at the attached ester groups, Figure S7: Programming results in a helix as temporary shape; the original shape was recovered at 55 °C. For better visualization of the effect, the hydrogel stripes were stained with Coomassi blue, as original hydrogel appear to be transparent, Table S1: Gel content (G) and volumetric degree of swelling (Q) at defined temperatures for different hydrogel compositions, Table S2: Calculated values for relative triple helix content (Χ_TH_) and relative single helix content (Χ_SH_), as determined by means of WAXS measurements, Video S1: Shape recovery of a sample at 55 °C programmed as helix (compare Figure S5).

## Abbreviations

DMSO	dimethyl sulfoxide
DMTA	dynamic mechanical thermal analysis
*ε*	strain
*E*	Young’s modulus
*E*′	storage modulus (determined by DMTA)
*E*″	loss modulus (determined by DMTA)
*ε*_b_	elongation at break
*G*	gel content
*G*′	storage modulus (determined by rheology)
GMA	glycidyl methacrylate
*H*	water uptake
OEG	oligo(ethylene glycol)
*Q*	volumetric swelling
*R*_f_	shape-fixity ratio
*R*_r_	shape-recovery ratio
SDS-PAGE	sodium dodecyl sulfate polyacrylamide gel electrophoresis
*σ*_max_	tensile strength
SMH	shape-memory hydrogel
SMP	shape-memory polymer
*T*	temperature
THF	tetrahydrofuran
*V*	volume
WAXS	wide-angle X-ray scattering
*X*_SH_	index of single helicity
*X*_TH_	index of triple helicity
*z*	thiol:methacrylate molar ratio
