# Supplementary material for: Thermally-Induced Shape-Memory Behavior of Degradable Gelatin-Based Networks
Source: Int J Mol Sci. 2021 May 31;22(11):5892. doi: 10.3390/ijms22115892 (PMC8197998; doi:10.3390/ijms22115892)
Supplement: Supplementary file 1 [file ijms-22-05892-s001.zip › ijms-1212887_Supp Info.pdf]

Supplementary Materials for

Thermally Induced Shape-Memory Behavior of Degradable Gelatin-Based Networks

by Axel T. Neffe[1], Candy Löwenberg[1], Konstanze K. Julich-Gruner[1], Marc Behl[1], and Andreas Lendlein[1,2]

1: Institute of Active Polymers and Berlin-Brandenburg Center of Regenerative Therapies, Helmholtz-Zentrum Hereon, 14513 Teltow, Germany

2: Institute of Chemistry, University of Potsdam, 14476 Potsdam, Germany

Table S1. Gel content (G) and volumetric degree of swelling (Q) at defined temperatures for different hydrogel compositions.

Sample ID[a]	*G* [wt%]	$Q^{4\ °C}$ [vol%]	$Q^{37\ °C}$ [vol%]	$Q^{55\ °C}$ [vol%]
G20_OEG1000(0.75)	86 ± 2	1180 ± 80	1110 ± 80	1030 ± 60
G20_OEG1000(1)	89 ± 2	1080 ± 30	1010 ± 50	940 ± 50
G20_OEG1000(2)	83 ± 2	1630 ± 90	1610 ± 120	1620 ± 30
G20_OEG1000(3)	71 ± 4	3050 ± 210	3050 ± 360	2680 ± 30
G20_OEG1500(0.75)	86 ± 1	1270 ± 90	1290 ± 80	1210 ± 60
G20_OEG1500(1)	87 ± 1	1260 ± 40	1290 ± 30	1250 ± 90
G20_OEG1500(2)	82 ± 2	1690 ± 70	1730 ± 70	1700 ± 80
G20_OEG1500(3)	77 ± 3	2950 ± 70	2960 ± 60	2950 ± 80
G20_OEG3400(0.75)	86 ± 1	1340 ± 40	1280 ± 40	1290 ± 130
G20_OEG3400(1)	81 ± 1	1620 ± 70	1630 ± 120	1640 ± 170
G20_OEG3400(2)	72 ± 2	2410 ± 150	2390 ± 200	2400 ± 130
G20_OEG3400(3)	42 ± 8	2890 ± 100	2970 ± 160	2900 ± 160

a: nomenclature: G20_OEG*y*(*z*), with G20 = 20 wt% concentration of gelatin in the crosslinking step, *y* = number average molar mass M_n of the OEG crosslinkers and *z* the thiol:methacrylate molar ratio.

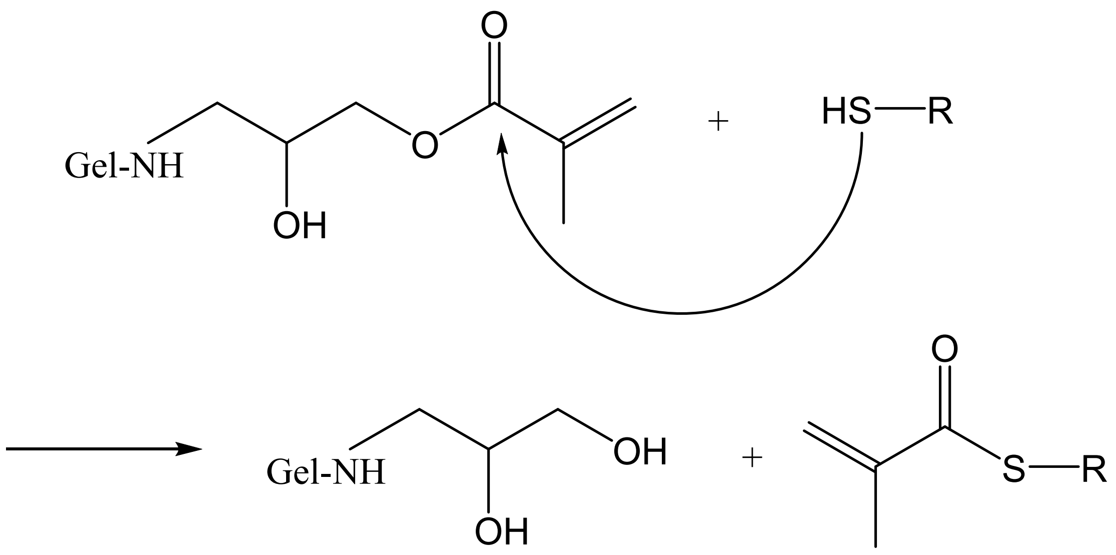

Figure S1. Thioester formation by reaction of a thiol with a glycidylmethacrylate group on GMA-gelatin as side reaction increasing in importance with increasing amount of thiols.

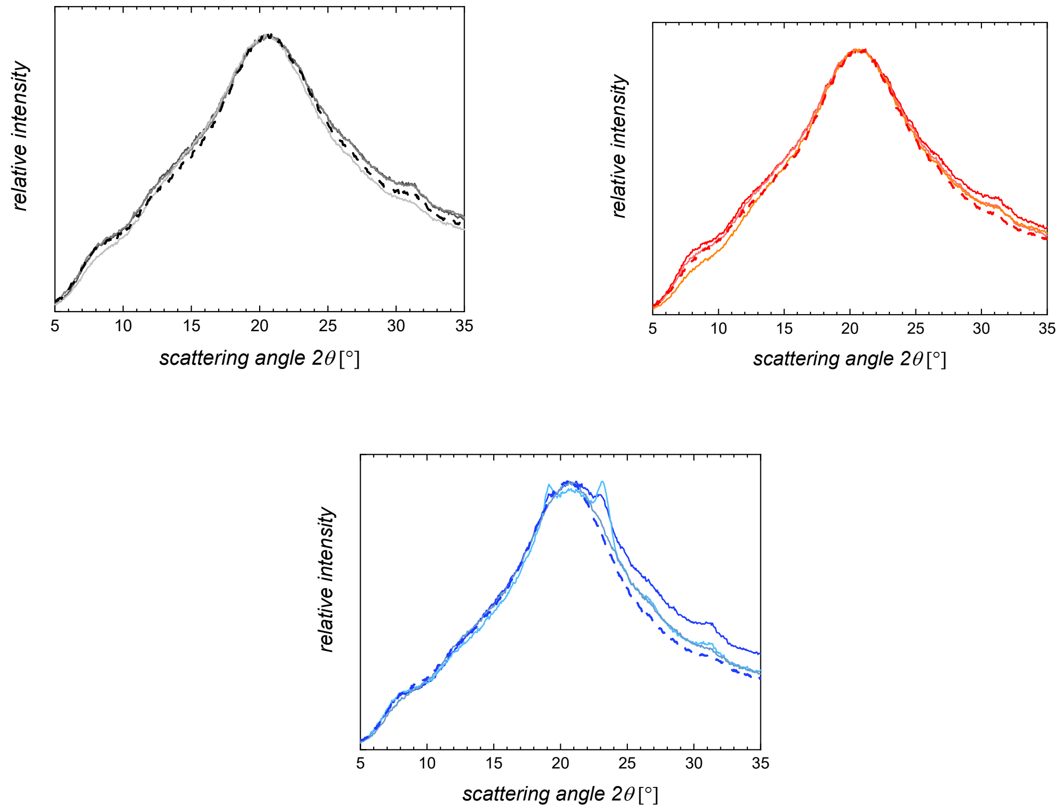

Figure S2. WAXS spectra of (A) G20_OEG1000(z), (B) G20_OEG1500(z), and (C) G20_OEG3400(z) networks. $z = 0.75$ (— — —), 1 (— — —), 2 (— — —), and 3 (-- -- --).

Table S2. Calculated values for relative triple helix content (X_{TH}) and relative single helix content (X_{SH}), as determined by means of WAXS measurements.

Sample ID[a]	X_{TH} [%]	X_{SH} [%]
G20_OEG1000(0.75)	4.6 ± 0.2	1.0 ± 0.1
G20_OEG1000(1)	3.9 ± 0.1	0.9 ± 0.1
G20_OEG1000(2)	4.4 ± 0.2	1.0 ± 0.1
G20_OEG1000(3)	4.3 ± 0.2	1.0 ± 0.1
G20_OEG1500(0.75)	4.5 ± 0.2	1.1 ± 0.1
G20_OEG1500(1)	4.0 ± 0.3	0.9 ± 0.1
G20_OEG1500(2)	4.4 ± 0.1	1.1 ± 0.1
G20_OEG1500(3)	4.5 ± 0.1	1.1 ± 0.1
G20_OEG3400(0.75)	6.1 ± 0.2	1.5 ± 0.1
G20_OEG3400(1)	5.6 ± 0.3	1.4 ± 0.1
G20_OEG3400(2)	5.8 ± 0.3	1.4 ± 0.1
G20_OEG3400(3)	6.0 ± 0.5	1.5 ± 0.1

a: nomenclature: G20_OEG*y*(*z*), with G20 = 20 wt% concentration of gelatin in the crosslinking step, *y* = number average molar mass M_n of the OEG crosslinkers and *z* the thiol:methacrylate molar ratio.

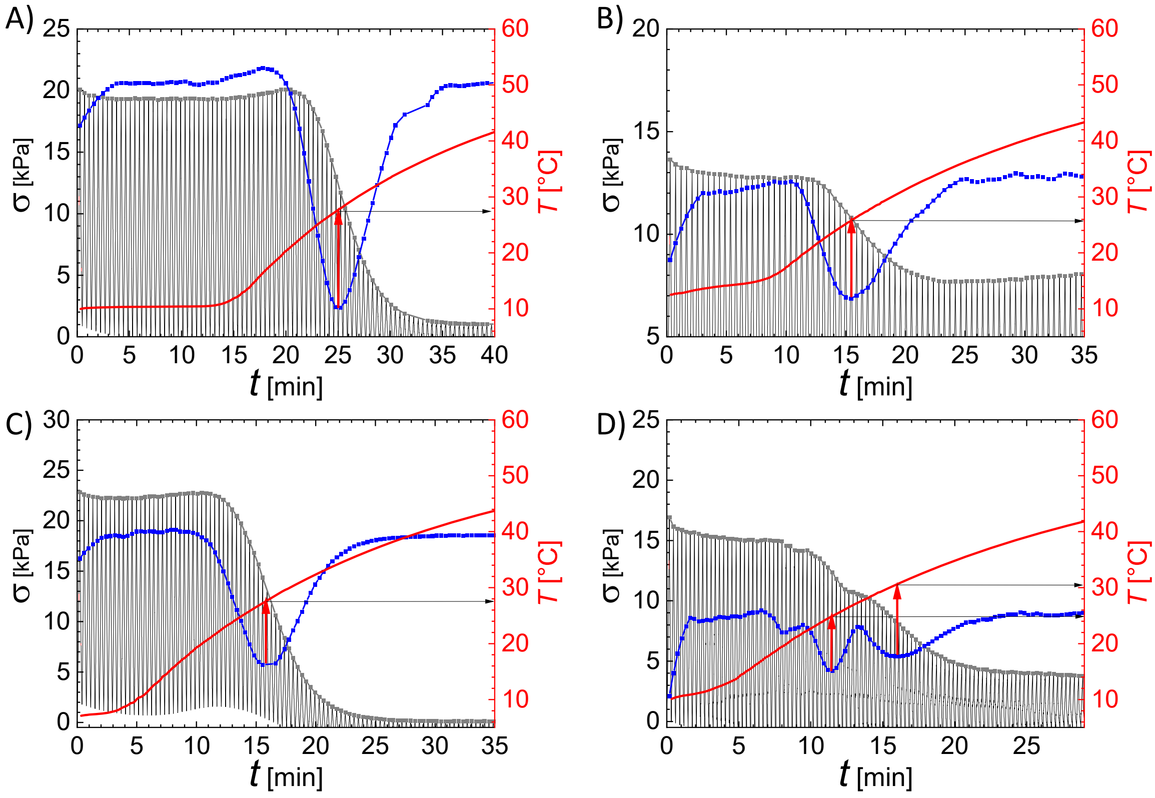

Figure S3. Determination of the transition temperature by rheological compression tests. A) G20_OEG1000(1), B) G20_OEG1000(2), C) G20_OEG3400(1), D) G20_OEG3400(2).

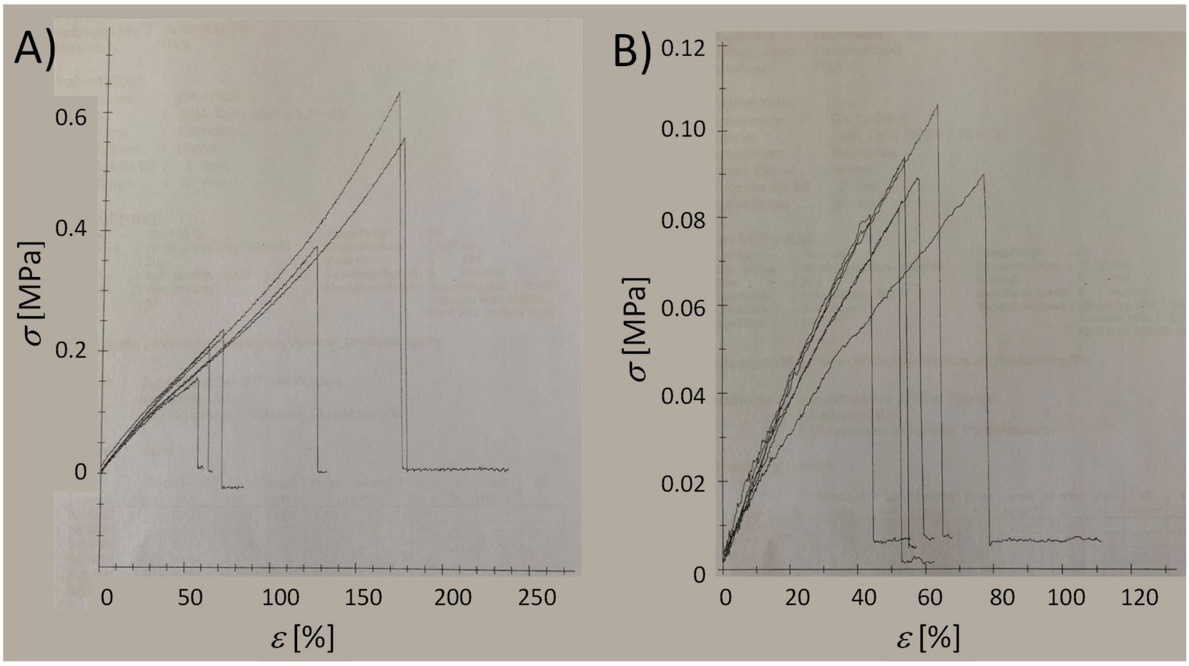

Figure S4. Tensile tests of G20_OEG1000(1) samples at 4 °C (A) and 55 °C (B). σ = stress, ε = strain.

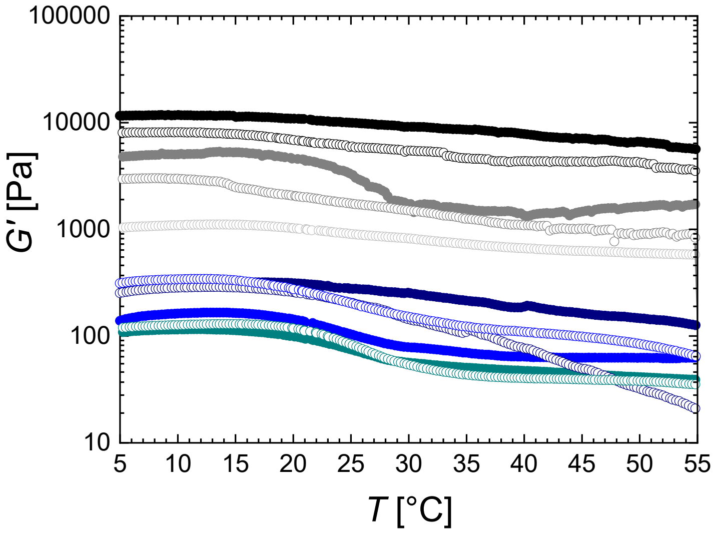

Figure S5. Rheological behavior of G20_OEG1500(1) hydrogels after 1(-●-), 1.5(-❍-), 2(-●-), 2.5(-❍-),3.5 (-❍-), 4 (-●-), 4.5 (-❍-), 5 (-●-), 5.5 (-❍-), 6.5 (-●-), and 6.5 (-❍-) days of hydrolytic degradation at 37 °C at pH = 7.4.

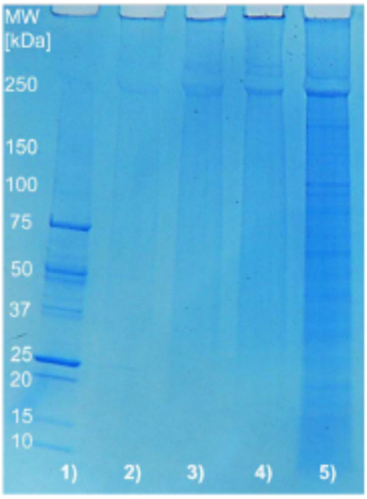

Figure S6. SDS-PAGE of the molar masses of the degradation products at t = 8 d: 1) standard, 2) G20_OEG1000(1), 3) G20_OEG1500(1), 4) G20_OEG3400(1). No larger gelatin fragments are observed, which suggests that hydrolysis primarily starts at the attached ester groups.

Method S1: SDS-PAGE

The determination of molar mass distribution of gelatin samples was carried out by SDS-PAGE in a Mini-Protean system (Bio-Rad, Feldkirchen, Germany), using 4-20% Ready Gels (Bio-Rad Laboratories, Feldkirchen, Germany). For the determination of the molar mass, a pre-stained SDS-PAGE standard was used. Gelatin samples were dissolved in distilled water at a concentration of 2 or 3 mg/mL. All samples were diluted 1:1 with a Laemmli sample buffer, containing 62.5 mM Tris-HCl, pH 6.8, 25% (w/v) glycerol, 2% SDS, 0.01% bromophenol blue, and 5% (w/v) 2-mercaptoethanol, followed by a heating to 90 °C for 5 min. Afterwards the samples were immediately cooled down to 25 °C. Then, 15 µL of each sample were loaded onto their respective lane in the gel and a current of 120 V was applied for approximately 45 min in running buffer (1L 10X buffer prepared with: 30.2 g Tris base, 144 g glycine, 10 g SDS). The protein bands were visualized after a 60 min staining (0.1 wt% comassie blue, 10 vol% acetic acid and 40 vol% methanol in water) and 60 min treatment in a destaining solution (10 vol% glacial acetic acid, 20 vol% methanol in water). The molar mass of the gelatin fragments was approximated by measuring the relative mobility of the standard protein molecular weight markers.

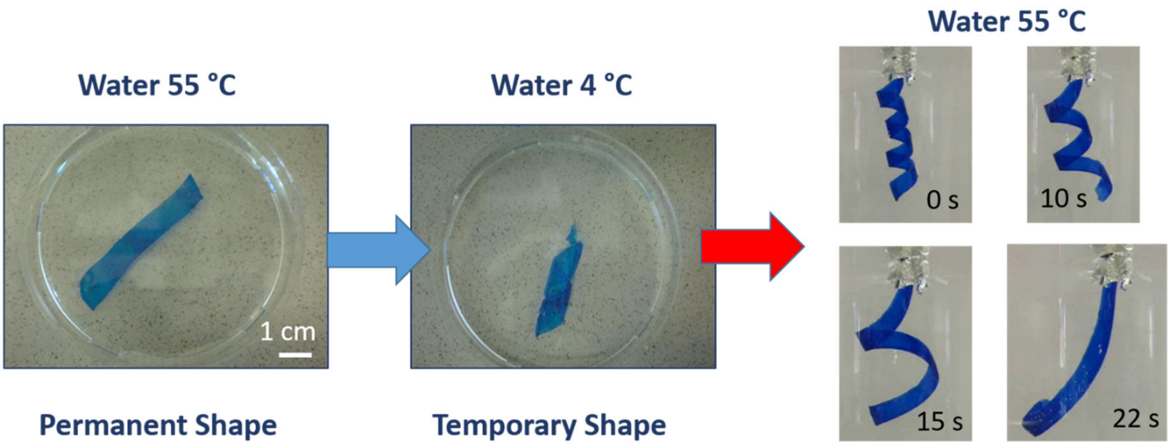

Figure S7. Programming results in a helix as temporary shape; the original shape was recovered at 55 °C. For better visualization of the effect, the hydrogel stripes were stained with Coomassi blue, as original hydrogel appear to be transparent.

Video S1. Shape recovery of a sample at 55 °C programmed as helix (compare Fig. S5).
